# Determination of short-chain fatty acids as putative biomarkers of cancer diseases by modern analytical strategies and tools: a review

**DOI:** 10.3389/fonc.2023.1110235

**Published:** 2023-06-27

**Authors:** Petra Chalova, Anton Tazky, Ludovit Skultety, Lenka Minichova, Michal Chovanec, Sona Ciernikova, Peter Mikus, Juraj Piestansky

**Affiliations:** ^1^ Department of Pharmaceutical Analysis and Nuclear Pharmacy, Faculty of Pharmacy, Comenius University, Bratislava, Slovakia; ^2^ Biomedical Research Center of the Slovak Academy of Sciences, Institute of Virology, Bratislava, Slovakia; ^3^ Toxicological and Antidoping Center, Faculty of Pharmacy, Comenius University, Bratislava, Slovakia; ^4^ Institute of Microbiology, Academy of Sciences of the Czech Republic, Prague, Czechia; ^5^ 2nd Department of Oncology, Faculty of Medicine, Comenius University and National Cancer Institute, Bratislava, Slovakia; ^6^ Biomedical Research Center of the Slovak Academy of Sciences, Cancer Research Institute, Bratislava, Slovakia; ^7^ Department of Galenic Pharmacy, Faculty of Pharmacy, Comenius University, Bratislava, Slovakia

**Keywords:** short-chain fatty acids, cancer, biomarker, mass spectrometry, separation methods, chromatography, capillary electrophoresis

## Abstract

Short-chain fatty acids (SCFAs) are the main metabolites produced by bacterial fermentation of non-digestible carbohydrates in the gastrointestinal tract. They can be seen as the major flow of carbon from the diet, through the microbiome to the host. SCFAs have been reported as important molecules responsible for the regulation of intestinal homeostasis. Moreover, these molecules have a significant impact on the immune system and are able to affect inflammation, cardiovascular diseases, diabetes type II, or oncological diseases. For this purpose, SCFAs could be used as putative biomarkers of various diseases, including cancer. A potential diagnostic value may be offered by analyzing SCFAs with the use of advanced analytical approaches such as gas chromatography (GC), liquid chromatography (LC), or capillary electrophoresis (CE) coupled with mass spectrometry (MS). The presented review summarizes the importance of analyzing SCFAs from clinical and analytical perspective. Current advances in the analysis of SCFAs focused on sample pretreatment, separation strategy, and detection methods are highlighted. Additionally, it also shows potential areas for the development of future diagnostic tools in oncology and other varieties of diseases based on targeted metabolite profiling.

## 1 Introduction

Short-chain fatty acids (SCFAs) are carboxylic organic acids with at least one to six carbon atoms. The main representatives of this group are formic acid, acetic acid, propionic acid, butyric acid, valeric acid, and caproic acid (1).

**Figure 1:**
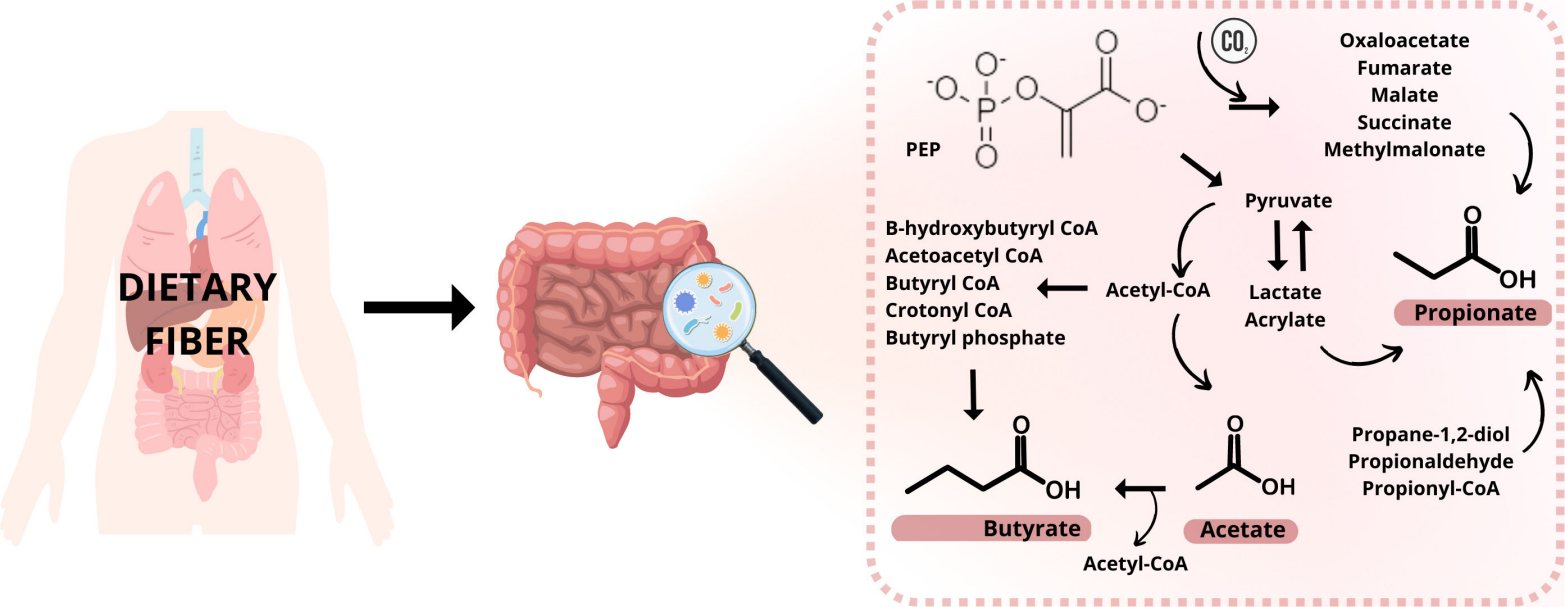
Production of SFCAs by gut microbiota (created in Canva Pro).

SCFAs are produced by microorganisms living in the gastrointestinal tract (gut microbiota, GM) by fermenting polysaccharides, oligosaccharides, proteins, peptides, and glycoprotein precursors in the colon (2, 3). This fermentation involves many reactions and metabolizing actions in the anaerobic microbial breakdown of organic matter leading to the generation of SCFAs, gases like CO_2_, methane, and H_2_, and heat (4). The procedure is schematically illustrated in **Figure 1**. The ability to produce caproic acid from lactate by gut microbiota and the production of valeric acid by the intestinal microbiota has also been suggested (5, 6). It is expected that 95% of all SCFAs are represented by the three main representatives – acetic acid, propionic acid, and butyric acid; while higher levels of acetic acid are relatively constant not only in the colon but also in the feces (1, 7). Butyrate and isobutyrate are the main energy source for colon cells. However, SCFAs are mainly produced in the GIT, they are able to affect a variety of diseases outside the gut. As a result of their impact on the immune system, they reduce the risk of inflammatory diseases, type II diabetes, cardiovascular diseases, obesity, and also cancer (8). There are various mechanism of action how these molecules affect the diseases. Typically, the effect is mediated via direct interaction with the responsible receptors, large variety of transport proteins or ion channels. For example, the inflammation and immune system are regulated by SCFAs due to reducing the number of immune cells such as macrophages, neutrophils, and dendritic cells and their migration, and differentiation of T and B cells (9–11). Two main mechanisms for SCFAs regulating inflammation are known. The first one is through cell signal transduction. The SCFAs are capable to bind cell membrane surface G protein-coupled receptors, namely FFAR2, FFAR3, GPR109A. Through the binding of these receptors, they could regulate different pathways (MAPK pathway, downstream NF-κB pathway) and concentration levels of Ca^2+^, and synthesis of cAMP. The second way is when SCFAs enter cells by passive diffusion and transport proteins (MCT1/4, SMCT1/2), they inhibit histone deacetylase and participate in the regulation of inflammation (12–15). In case of cardiovascular diseases, the mechanism of action depends on the type of the disease. Reduction of the blood pressure in hypertension is achieved by mechanisms such as down regulation of the renin-angiotensin-aldosterone system, restoring the decreased expression of GPR43 and GPR109A, and inducing vasodilatation via olfactory receptor – Olfr78 and GPR41 (16, 17). In the case of atherosclerosis and its prevention, SCFAs are regulating the production of regulatory T (Treg) cells and suppress histone deacetylases and they could decrease total low-density lipoprotein (LDL) and very-low-density lipoprotein (VLDL) cholesterol levels (18–20). SCFAs are also directly involved in glucose synthesis and so contribute to the control of the glucose homeostasis. SCFAs can directly stimulate the goblet cells in the colon to secrete glucagon-like peptides (GLP-1) by binding the free fatty acid receptor FFAR2 on the cell membrane (21) or they can directly promote pancreatic beta-cell proliferation by binding FFAR2 (22).

Moreover, there is a potential for these molecules to serve as putative biomarkers of various diseases emergence and progression. Identification and quantification of such molecules in biological samples are of great clinical importance. It is due to the possibility to assess early disease diagnosis, its progression, drug response, disease prevention, and drug target identification (23). Thus,

the development of appropriate analytical methodologies for the accurate identification and determination of SCFAs levels in biological matrices is an important task in the field of recent research.

## 2 SCFAs as biomarkers of various diseases

The term biomarker is a subcategory of the medical sign just like objective indications of the medical state of the respondent, which may be measured accurately and reproducibly by various methods. Biomarkers play a critical role in drug development strategies and in understanding the relationships between measurable biological processes and clinical outcomes (24). Biomarkers in practice include tools and techniques that can be helpful in the prediction, cause, diagnosis, progression, regression, or outcome of treatment of disease. Currently, there is a growing interest in the direct measurement of those markers that actually point to the causal path of the disease (25). Some biomarkers (26) are already effectively used in the diagnosis and therapy of cardiovascular diseases (e.g. copeptin, galectin-3, cardiac troponins) (27), infections (e.g. pentraxin) (28), immunological and genetic disorders (e.g. neurexin 1 deletion, SHANK 3) (29), or cancer [e.g. carcinoembryonic antigen (30), alpha-fetoprotein (31)].

Biomarkers in relation to therapeutic response can be dynamic or static. Dynamic biomarkers describe the progression and associated treatment response, and they are widely used in patient care or drug development. A good example of a dynamic biomarker is prostate-specific antigen (PSA) for response to prostate cancer treatment (32). Static biologic markers are just prognostic and predict a clinical response (33). Biomarkers can be also classified according to their chemical structure. We distinguish simple molecules such as metabolites, carbohydrates, steroids, and lipids, but also more complex molecules like peptides and proteins – e.g., insulin, hemoglobin A, hemoglobin C, PSA, or C-reactive protein – CRP (34).

**Figure 2:**
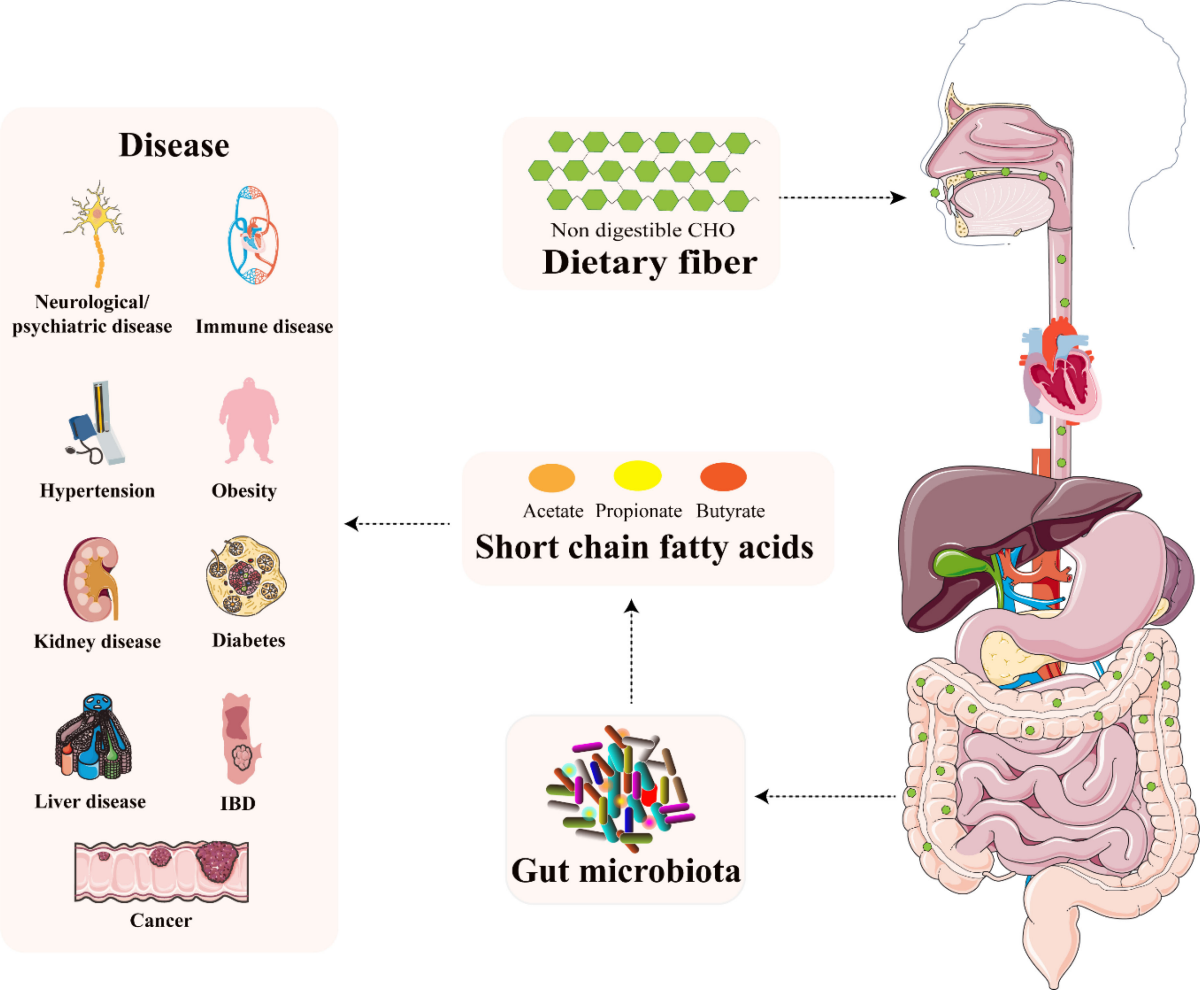
The relationship between microbiota-derived SCFAs and several diseases. Reprinted and modified with permission from (37).

Recently, an increasing interest is devoted to the SCFAs (35, 36). It is due to the fact that SCFAs not only influence the signal transduction pathway in the gut, but they also reach tissues and organs outside of the gut, through their circulation in the blood (37) which is schematically illustrated in **Figure 2**. Their role in gastrointestinal (GIT) microbiota disturbances, irritable bowel syndrome (IBS), or autism spectrum disorders (ASD) was documented (34, 38). Decreased levels of SCFAs were investigated in inflammatory bowel disease (IBD) patients and rat models and it was shown that supplementation by butyrate was an effective tool in attenuating colitis (39–41). A study by González-Hernández et al. (42) revealed the valuable potential of SCFAs in comparison between HIV-positive and HIV-negative population. It was demonstrated that HIV-positive patients were characterized by higher levels of total SCFAs (mainly augmented propionic acid values), compared to HIV – negative subjects.

The association between SCFAs in the serum and patients with multiple sclerosis was also confirmed (43, 44). Levels of propionate were lower in patients with multiple sclerosis than in the serum of healthy controls. In this study, a positive correlation of circulating T follicular regulatory cells and T follicular helper cells with propionate serum levels was demonstrated. Moreover, levels of butyrate were associated positively with frequencies of IL-10-producing B-cells and negatively with frequencies of class-switched memory B-cells. TNF production by polyclonally activated B-cells correlated negatively with acetate levels (44).

In particular, the gut microbiota has been shown to provide a reservoir of biomarkers that assist in the early detection and treatment of cancer diseases (45, 46). The gold-standard method such as colonoscopy, biopsy, or use of contrast labels are screening techniques with high invasiveness and costs. According to novel studies, different types of carcinomas could be detected by SCFAs in the fecal, serum, plasma, tissues or semi-skimmed milk samples as oncological markers by analytical methods with high sensitivity and precision (47, 48). An excellent example is a colorectal carcinoma where the implementation of custom-made methods with minimal invasiveness, protection, and reproducibility are highly desirable. Therefore, the measurements of metabolites in living systems as a reaction to pathophysiological influences, genetic modification, or environmental factors are of great benefit (49).

As can be seen, the aforementioned examples of changed SCFAs levels in various diseases clearly documented the promising potential of SCFAs to be suitable markers of disease identification, disease specification and monitoring of therapeutic intervention. However, there is still the need to perform specific trials which will confirm the real potential of these molecules to be reliable biomarkers implementable into the routine clinical praxis. Recently, the greatest potential of SCFAs as biomarkers can be seen in the field of oncological diseases. It is due to the fact that various types of cancer are very closely accompanied with diet regime and gut microbiome. The very close relation between gut microbiome and its metabolites with human health can therefore bring answers on the important questions such as how affect the microbiome and its metabolites the disease and how the disease affect the microbiome and levels of its specific metabolites.

## 3 SCFAs in oncological diseases

Many oncological diseases are associated with the gut microbiota, especially bacteria producing SCFAs (50), however, the exact mechanisms of action are still not well understood and exactly explained. It is suggested that the anticancer activity of the SCFAs is mediated via multiple molecular pathways resulting in the inhibition of cancer cell proliferation and induction of apoptosis in cancer cells (51). In the case of colon cancer, these effects are very closely associated with the initiation of histone hyperacetylation in cancer cells (52). Moreover, SCFAs are able to recognize G-protein-coupled receptors, such as GPR41, GPR43, or GPR109A which are situated on the surface of macrophages and T cells, and their activation leads to enhanced numbers of regulatory T cells and levels of the anti-inflammatory cytokines. Asarat el al (53). showed in their study performed on human peripheral blood mononuclear cells that the interleukin-10 (IL-10) levels increased in acetate, propionate and mixed SCFAs but not in butyrate and slight reduction of transforming growth factor-ß (TGF- ß) was associated with mixture of SCFAs. Further investigation led to the discovery that the synthesis of the polyamines were upregulated and the ornithine decarboxylase (an enzyme of polyamine metabolism) is responsible for the inhibition of cancer cell proliferation (54). It has been demonstrated that especially butyrate plays a crucial role in the potential anticancer activity in various cancer diseases. It was confirmed that butyric acid strongly promoted histone crotonylation in HCT116 colon cancer cells (55). This investigation is in a good agreement with several studies that suggest the associations between SCFAs and post-translational modifications of histones – crotonylation, bytyrylation, and propionylation, namely. These modifications are similar to histone acetylation. *In vitro* studies suggest that histone crotonylation may be highly regulated over the cell cycle and deregulation of this process may result in cancer development (56).

Cancer metabolomics is an active and robust field. The metabolome of the oncological patient could provide insight through the host-microbiome interactions and may result in the understanding of the pathogenesis of disease based on the metabolic activity of bacterial communities (57). There is increasing evidence that SCFAs may modulate inhibitor responses in humans. SCFAs are known to be key regulators of various immune functions and therefore we can use them as promising predictive biomarkers of immunotherapy efficacy and oncological diseases (58).

### 3.1 Breast cancer

In 2021, He et al. (59) published a study dealing with the comparison of the composition and symbiosis of intestinal microbiota between healthy premenopausal women and premenopausal breast cancer patients. It was demonstrated that the group of premenopausal women with breast cancer was characterized by a significant reduction of the abundance of some SCFA-producing bacteria such as *Pediococcus*, *Fusobacterium*, and *Enhydrobacter*. The study presented the fact, that more than 70% of all investigated SCFAs were represented by acetic, propionic, and butyric acid. Moreover, the levels of these three SCFAs were significantly reduced in the case of premenopausal women with breast cancer. The levels of other investigated SCFAs were also decreased, but no statistical significance was confirmed. SCFAs are also produced by *Pediococcus* and *Fusobacterium*, and the study by He et al. (59) demonstrated that their significant decrease in patients with breast cancer caused a significant decrease of SCFAs in the intestine of subjects. The authors have also investigated the inhibition effect of propionate and butyrate on breast cancer cell lines (SKBR3, MCF7, BT-20, BT474, MDA-MDB-231) viability. The main anti-cancer effect was observed in the case of butyrate, and it was shown that its increasing concentration led to improved effect strength. The obtained results indicate that SCFAs, especially butyrate, may play a certain role in alleviating the progression of breast cancer cells.

Zhu et al. (60), performed untargeted metabolomics, targeted metabolomics (SCFAs), and 16 S rDNA sequencing studies on fecal samples from 14 breast cancer patients and 14 healthy volunteers. The SCFAs content in fecal samples was analyzed quantitatively. The analysis included acetic, propanoic, isobutyric, butyric, isovaleric, and pentanoic acids. Results from this study revealed that the levels of acetic, propanoic, butyric, and isovaleric acid were lower in the breast cancer patient group in comparison to the control group. On the contrary, levels of isobutyric and pentanoic acid increased. Based on 16S rDNA sequencing, alterations in the gut microbiota of breast cancer patients were determined. The abundance in the genus of *Lautropia*, *Rothia*, *Centipeda*, *Corynebacterium*, *Anaeroglobus*, *Actinomyces*, *Selenomonas*, *Fretibacterium*, and *Tannarella* increased in the cancer group in comparison to the controls. Further, the correlation between microbiota and SCFAs profile was performed, and a weak association was observed. A correlation (p < 0.5) between genera of *Lautropia*, *Rothia*, *Corynebacterium*, *Anaeroglobus*, *Actinomyces*, *Selenomonas*, *Fretibacterium*, and pentanoic, and isobutyric acid was investigated. On the other hand, acetic and propionic acids were correlated with the genus of *Subdoligranulum* (p < 0.5). The results indicate that the imbalance of gut microbiota in breast cancer patients might lead to metabolic disorders.

**Figure 3:**
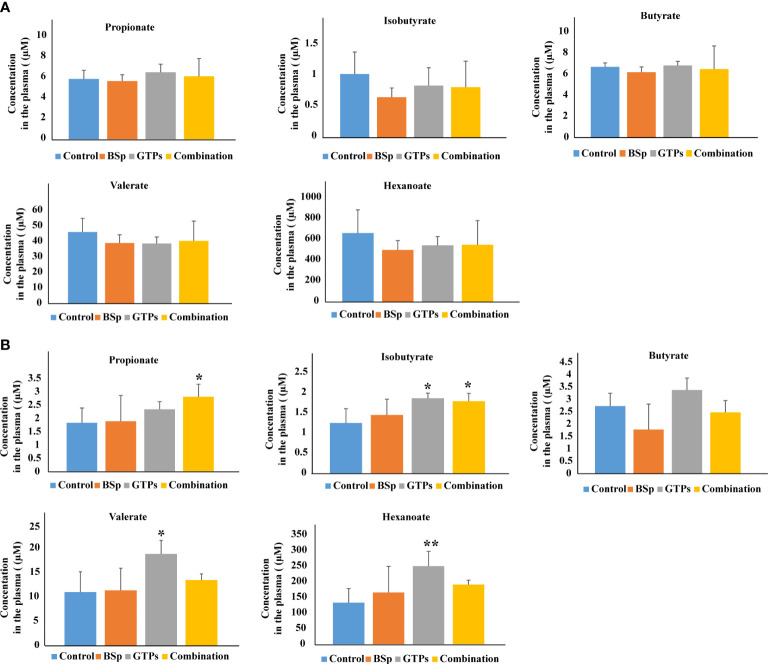
Plasma levels of SCFAs analyzed by LC-MS in BSp-fed, GTPs-fed, and combination-fed mice and control-fed mice. **(A)** SCFAs concentrations in dietary treatment mice versus control treatment in BE group. **(B)** SCFAs concentrations in dietary treatment mice versus control treatment in LC group. *p < 0.05, **p < 0.01, ***p <0.001. Reprinted with permission from (61).

The nutritional impact on the gut microbiota and plasma SCFA in the mammary cancer was investigated by Sharma et al. (61). The experiment was performed on the Her2/neu estrogen receptor-negative, ER (-), transgenic mice and the effect of broccoli sprouts (BSp), green tea polyphenols (GTPs) and their combination was evaluated. The exposure periods were beginning in early life (BE) and lifelong from conception (LC). In both treatments, the combination group achieved maximum efficacy in delaying mammary tumor volume and delay in tumor latency. The GTPs group was more efficient in comparison with the control group. In this diet group, plasma levels of SCFAs (isobutyrate, valerate, and hexanoate) were increased. The combination of the diet was accompanied with increased levels of propionate and isobutyrate. The combination of the dietary treatment mice versus controls in BE or LC group is compared in **Figure 3**. The authors of the study hypothesize that increased SCFAs in GTPs diet may induce epigenetic modification resulting in a decrease in tumor volume and an increase in tumor latency in the LC group. Moreover, these findings suggest that various time windows associated with dietary treatments during the adult lifespan have a profound effect on gut microbiota establishment and SCFAs profiles, and might represent a potential intervention for ER (-) breast cancer in women (61).

Interestingly, this study realized by Sharma et al. (61) gave opposite results as the study by Zhu et al. (60) in case of isobutyrate. The differences in the results may be caused by the type of samples used in the studies, i.e. mice model *vs*. humans and also in the whole experimental setup. Moreover, relatively small number of subjects were included into the studies. Therefore, there is the need to confirm the results of both pivotal studies by appropriate studies with suitable number of investigated subjects with exact characterization and stratification.

### 3.2 Colorectal cancer

Chronic inflammation is the major risk factor for the development of colorectal cancer (CRC) (62). Improved incidence and mortality in patients with inflammatory bowel disease (IBD) were reported (63, 64). Recently, a large variety of studies is dealing with the anti-inflammatory properties and immunomodulatory effects of SCFAs and their antitumor action in the case of CRC (52, 65–68). *In vivo* studies in animal models (mice), showed that the incidence, number, and size of CRC tumors were significantly reduced in the mice treated with SCFAs mixture. A similar positive effect was also observed in mice with adenoma and adenocarcinoma undergoing the same treatment (69). The vast majority of the studies suggest that butyrate is pivotal for maintaining colon health and CRC suppression through dietary fiber (40, 70–72). On the other hand, the cellular effects of butyrate are complex, and therefore not only positive but also negative influences on cell proliferation, differentiation, and apoptosis were observed (73). It means that butyrate presents both anti- and pro-carcinogenic activities, what is known as the “butyrate paradox”. The final effect, i.e., provoking or preventing cell proliferation depends on time, cell type, and butyrate concentration (74). Typically, carcinogenesis is induced by a low concentration of butyrate and its higher concentrations are responsible for the inhibition effect on tumorigenesis and tumor progression by the histone hyperacetylation-mediated pathway through conversion of inactive procaspase-3 to catalytically active protease (apoptotic) (51, 75–77).

Similarly, anti-inflammatory and antitumor activity were also observed in patients in the case of a sodium salt of butyrate (NaB) (78, 79). The novel study by Xi et al. (80) showed that NaB decreased the proliferation ability of CRC cells in a dose- and time-dependent manner. If the NaB concentration increased, the number of apoptotic CRC cells increased too. The CRC cells were apoptotic because of the block in the G phase of the cell cycle and suppression of invasiveness and migration capabilities. Moreover, there was identified a large variety of mRNAs and lncRNAs at different expression levels involved in the CRC inhibition by NaB. Especially, three differentially expressed mRNAs – i.e., HMGA2, LOXL2, and ST7, significantly correlate with the prognosis of CRC (80).

Liu et al. (81) revealed that NaB could inhibit colorectal carcinoma. The tumor size was significantly reduced in NaB treated mice groups in comparison to the azoxymethane and dextran sulfate sodium salt (AOM/DSS) mice group. Treatments were performed by intraperitoneal injection (*i.p.*) and by oral administration (*p.o.*) of NaB. The route of administration was also necessary for the final antitumor activity. The average number of tumor was lower in the case of *i.p.* NaB application (81). Previous research revealed that oral intake of NaB inhibit DSS-induced colitis in a mouse model, while *i.p.* injection of NaB leads to reducing inflammation. Marked sensitivity of mice to DSS, may be caused by oral intake of NaB, which could result in the production of inflammatory factors in the lamina propria dendritic cells (82). In addition, the NaB administration improved the immunity of AOM/DSS-treated mice and therefore it may inhibit the progression from chronic colitis to colorectal carcinoma (81). However, the results may be affected by the animal model used in the experiment. Carcinogen-induced, genetically engineered, or transplant models are typically used in CRC studies.

In the studies of circulating SCFAs and their associations with CRC, adenomas and high grade dysplasia, levels of seven SCFAs were measured (acetic acid, propionic acid, i-butyric acid, butyric acid, 2-methylbutyric acid, i-valeric acid, valeric acid). In the case of CRC patients in comparison with controls, the levels of SCFAs were as follows: acetic acid – 164 µM for the control group and 198 µM for CRC group, propionic acid – 14 µM for the control group and 11 µM for CRC group, i-butyric acid – 8.8 µM for the control group and 9.5 µM for CRC group, butyric acid – 9.8 µM for the control group and 10.18 µM for CRC group, 2-methylbutyric acid was 13 µM in the control group and 14 µM in the CRC group, i-valeric acid – 18 µM in the control group and 17 µM in the CRC group, valeric acid – 4.1 µM in the control group and 3.9 µM in the CRC group. The significant changes were in the levels of acetic acid (increase), propionic acid (decrease), i-butyric acid (increase), and butyric acid (decrease) (83).

### 3.3 Lung cancer and melanoma

The importance of SCFAs as putative cancer biomarkers was also presented in the case of non-small lung cancer and melanoma. Botticelli et al. demonstrated that a group of non-small cell lung cancer patients with early progression was characterized by low levels of some SCFAs (propionic, butyric, acetic and valeric acid). This group of patients showed also lower levels of some amino acids, but higher levels of alkanes, methyl-ketones, and p-krezol (84).

Lung cancer and gastrointestinal (GIT) tumors (especially pancreatic tumors) are the main causes of cancer cachexia (85). In a recent study, total SCFAs concentration in faeces was measured in healthy controls and cachectic and non-cachectic patients with pancreatic and lung cancer. The results clearly showed lower levels of total SCFAs in cachectic patients compared to non-cachectic ones. The most significant decreased analyte was acetate. Interestingly, a comparison of lung cancer cachectic patients with pancreatic cancer cachectic patients showed significantly reduced concentrations of butyrate and acetate only in pancreatic cancer patients (86).

According to a study of lung cancer and the anticancer effect of sodium propionate (SP), the treatment with SP led to significantly lower cell growth than in treated control cell lines (87). These results support the previous studies, where was shown that SCFAs may suppress the growth of cancer cell lines (88). In the actual study, the SP activity leads to the inhibition of lung cancer cell proliferation by inducing cell cycle arrest (G2/M phase). Different expression levels of cycle-related genes after SP treatment were also identified. The authors hypothesize that the SP treatment may affect the whole cell cycle machinery, particularly G2/M arrest, and this may result in the inhibition of cell proliferation. Even though the SP treatment is usually known in colon cancer therapies, the author claims that SP may be applicable for the therapy of lung cancer patients like an anticancer drug (87).

It is expected that the gut microbiota composition is associated with antitumor efficacy in patients with metastatic melanoma treated with anti-CTLA-4 and anti-programmed cell death protein-1 (anti-PD-1) monoclonal antibodies (mAbs) (89). Coutzac et al. presented the possibility of SCFAs as markers of clinical outcome. Higher blood levels of propionate and butyrate were associated with resistance to CTLA-4 blockade and a higher number of regulatory T (Treg) cells. The obtained results clearly showed the potential of propionate and butyrate serum levels to be used as an indirect systematic marker of the microbiota composition associated with clinical outcomes in patients treated with ipilimumab (90).

A study focused on the melanoma cancer cells ability to metastasize in the lungs showed that feeding mice with probiotics (VSL#3, Ferring Pharmaceuticals) could affect tumor progression in the extra-intestinal tract. The authors used B16F10 cancer cell line with high capability to metastasize to the lung and different treatments by probiotics. Results of this study indicate that feeding mice with probiotics (VSL#3) attenuate the lung metastasis of melanoma cells and the survivability of mice was prolonged. They also monitored the levels of SCFAs in the intestine and serum of mice using GC-MS after 7 and 14 days of the treatment by probiotics. Significantly increased concentrations of acetate, propionate, and butyrate were observed after the treatment (91). Zhou et al. (91) made other studies to explore how SCFAs levels are associated with the suppression of melanoma cell lung metastasis. It was shown a decrease in tumors after butyrate and propionate treatment, but not after acetate treatment. These data declare that propionate and butyrate were the main SCFAs associated with the inhibition of melanoma cell metastasis.

### 3.4 Prostate cancer

Lifestyle, especially diet, and its changes linked to the gut microbiome play an important role in prostate carcinogenesis as well (92). Typically, western-style diets are the major risk factors for prostate cancer (93). SCFAs regulate prostate cancer growth via systemic and local prostate insulin-like growth factor-1 (IGF-1). IGF-1 is secreted mainly from the liver and muscles and plays an important role in the development of bones and muscles. IGF-1 is also secreted from prostate cancer cells in an autocrine manner and enhances prostate cancer growth via the mitogen-activated protein kinase (MAPK) and phosphatidylinositol-3 kinase (PI3K) signaling pathways (94). In patients with benign prostatic hyperplasia, higher faecal levels of isobutyric and isovaleric acid were observed in comparison to healthy controls (95). Similarly, in another study, the relative abundances of SCFA-producing bacteria (i.e., *Rikenellaceae*, *Alistipes*, *Lachnospira*) were significantly increased in a cohort of patients with high-risk prostate cancer (96). Moreover, some studies using human cell lines have presented the putative inhibitory effect of butyric and propionic acid on prostate cancer (97, 98).

## 4 Analysis of SCFAs

**Figure 4:**
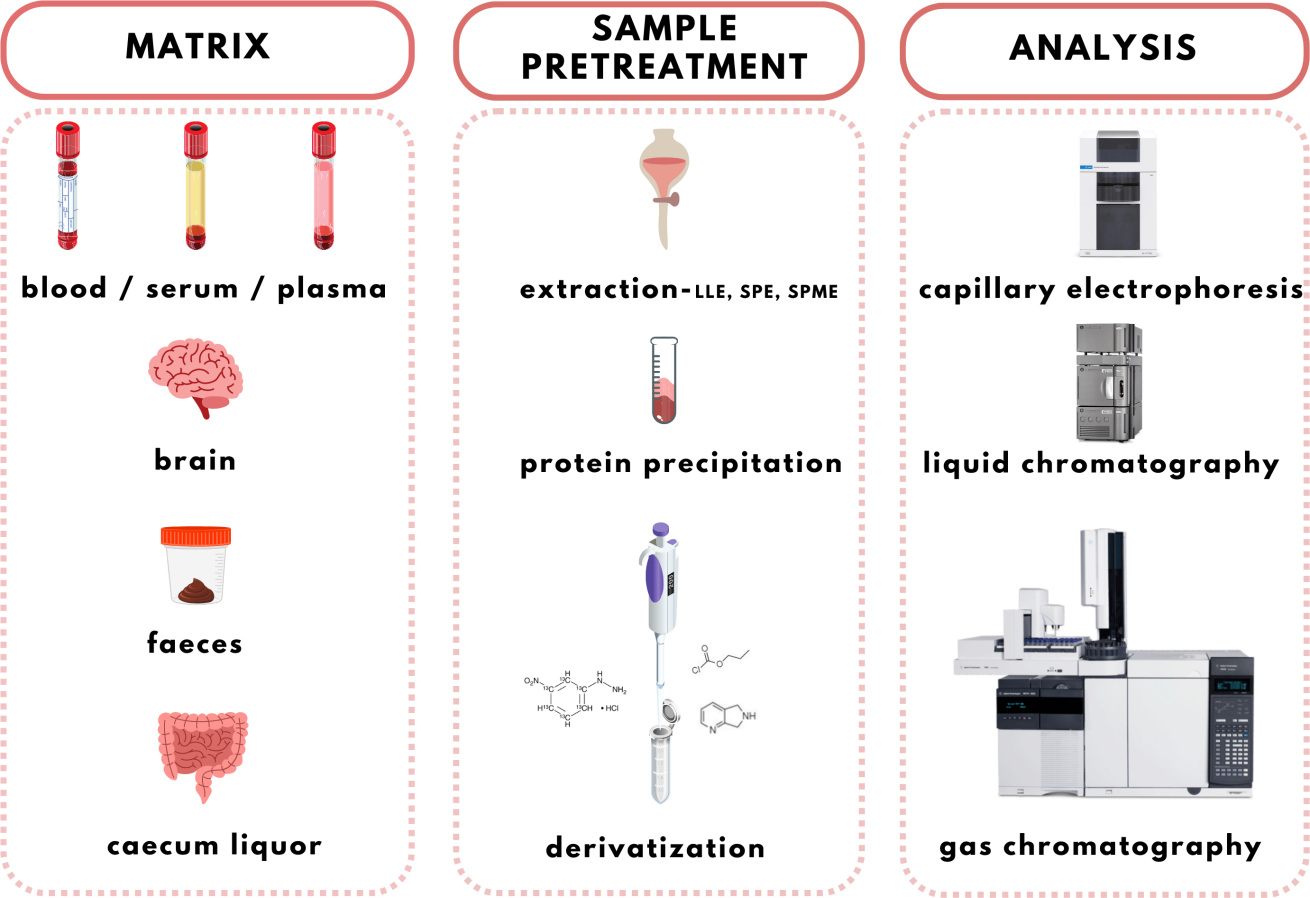
Analytical workflow of the SCFAs analysis (created in Canva Pro).

The increasing evidence about the important role of SCFAs in health and homeostasis is the reason for the development and discovery of new qualitative and quantitative analytical methods applicable in clinical laboratories. Several methods have been used to analyse SCFAs in biological samples including gas chromatography (GC), high-performance liquid chromatography (HPLC), or capillary electrophoresis (CE) coupled with various detection techniques, such as ultraviolet (UV) spectrophotometry, flame ionization detection (FID), and mass spectrometry (MS). The use of an appropriate analytical method depends on the sample type taken into the analysis and also on the purpose of the analysis (7, 99). GC-FID and GC-MS are the most used tools (100). However, improved interest is dedicated to the alternative approaches based on HPLC-MS and CE-MS recently. Even though MS is a highly advanced technology characterized by high sensitivity and selectivity when coupled with separation methods (especially chromatographic) the limitations for SCFAs detection still exist. Direct analysis of SCFAs in biological samples is challenging due to their poor ionization and unsuitable chromatographic properties. Therefore, it is necessary to improve the separation process and MS detection using appropriate sample treatment procedures. Moreover, MS approaches offer several options for the analysis of volatile compounds with high accuracy, and sensitivity which makes them suitable for the SCFAs analysis in various types of samples (101, 102). A complex overview of the SCFAs analysis workflow is illustrated in **Figure 4**.

### 4.1 Sample type and sample pretreatment

For clinical purposes, SCFAs have been measured in various biological materials such as blood plasma or serum, brain, equine caecum liquor, and especially faeces (103–108). The vast majority of papers are focused on the analysis of faeces or faecal water, because it is suggested that this non-invasive sample type reflects best the linkage of gut microbiota to pathological conditions (52, 57, 109, 110). Typically, the samples should be frozen after the collection and stored at -80°C (7). Interestingly, the faecal samples should not be lyophilized, because this procedure decreases the levels of SCFAs (111). Some studies used the following faecal sample storage procedure: dilution (or extraction) of the sample with 10% glycerol, cooling to 4°C, snap freezing within 24h, and final storage at -80°C (109, 112).

Recently, the interest of researchers is more focused on the analysis of SCFAs in blood samples (i.e., plasma, serum). It is due to the assumption that the SCFAs blood levels can reflect the development and/or progression of the pathology (especially cancer diseases). Moreover, approximately 95% of SCFAs are absorbed from the colon, so the complex view of the influence of microbiota-associated metabolites on an organism demands the quantification of these molecules both in faeces and in the blood, however according to the literature the content of the colon represents a better option (90, 99, 113, 114). The blood samples should be processed as soon as possible after the blood collection and the resulting plasma or serum sample should be stored at -80°C. Such an approach is relatively safe because it minimizes the risk of analyte alterations in the sample. Before the analysis, the plasma samples are typically thawed at 4°C (115).

Before the analysis of the SCFAs, it is necessary to prepare the sample appropriately. Various sample pretreatment strategies are recommended to overcome the problems with high volatility, polarity, or thermostability of the analytes. However, the GC-MS method can be used for samples without pretreatment, but it causes the loss of SCFAs and lower recoveries. Therefore, the achievement of concentrated and clean analytes is one of the main goals of the sample preparation procedure. Typically, several pretreatment approaches are used before SCFAs analysis, i.e., liquid-liquid extraction (LLE), solid-phase extraction (SPE) and solid-phase microextraction (SPME), protein precipitation, and derivatization.

#### 4.1.1 Liquid-liquid extraction

Liquid-liquid extraction (LLE) is a common sample pretreatment technique. The extraction procedure is based on the use of two phases – aqueous (sample) and organic (represented by organic solvents, e.g., chloroform, ethyl acetate, ether, etc.). Such an experimental environment allows the partitioning of the analyte between the aqueous sample and the added organic solvent (116). The main advantage of the LLE is the possibility to perform direct quantitative measurements right after the extraction. On the other hand, LLE is a non-eco-friendly and time-consuming procedure. However, the knowledge about the use of appropriate extraction solvents has accumulated in the literature over many decades, which significantly facilitates the choice of organic solvent, pH, and type of various reagents, which can be used for sample clean-up and so lead to the development of methods with high extraction yields (117).

The partially hydrophilic nature of SCFAs makes their quantitative extraction to hydrophobic organic solvents difficult. Therefore, the approaches based on acidification of the sample are beneficial because such procedure keep acids less hydrophilic and thus facilitates the extraction (7). A study performed by García-Villalba et al. (118) focused on the optimization of the LLE procedure of SCFAs from faecal samples. Three different solvents were investigated – diethyl ether, dichloromethane, and ethyl acetate, namely. However, ethyl acetate and diethyl ether showed similar extraction efficiencies, finally, ethyl acetate was selected due to the handling difficulties and extreme flammability of diethyl ether. Acidification with orthophosphoric acid during the extraction procedure was also beneficial leading to improved extraction of SCFAs. The proposed extraction protocol was simple, fast, and inexpensive. In one hour, it was possible to extract about 30 samples. Moreover, the consumption of organic solvent was low (1 mL per sample) and only 100 mg of the sample was used.

#### 4.1.2 Protein precipitation

Protein precipitation is a widely used sample pretreatment procedure in the analysis of biological fluids. It is used to separate proteins from the solution and so to decrease interferences of demanded substances with them. The process of precipitation must be quick, and scalable, and small volumes of solvents should be used. The protein precipitation procedure is often used in SCFAs analysis in serum or plasma due to the rapid sample processing and possible direct analysis depending on the analytical technique used. This approach was presented by Rahman et al. who performed a simple supernatant transfer after the precipitation step and direct GC-MS analysis (119).

Precipitation of proteins in serum samples was effectively performed by meta-phosphoric acid (96) or by cold isopropanol (120). In the case of cell culture samples, 16% (w/v) trichloroacetic acid was used as a suitable precipitation agent (121). A possibility of plasma samples deproteinization represents 5-sulfosalicylic acid solution (122). For faecal samples, a large variety of organic solvents is available, such as acetonitrile, methanol-chloroform (3:1, v/v), acetonitrile-chloroform (3:1, v/v), or methanol (123).

#### 4.1.3 Solid-phase extraction and solid-phase microextraction

Solid phase extraction (SPE) is often used as a suitable sample preparation method for a large variety of biological samples. Recently, novel environmentally stable conducting polymer materials are used for SPE. For the SCFAs extraction and concentration were developed polyacrylonitrile-poly(3,4-Ethylenedioxythiophene)-PAN/PEDOT nanofibers which were a part of SPE preparation columns (124). For the preparation of SCFAs samples was also used SPE segmental elution procedure (125).

Solid phase microextraction (SPME) is a green and solvent-free method for pretreatment of samples. SPME enriches and reserves analytes for further analysis by exposing the extraction fiber over the aqueous surface, or by embedding it into the aqueous matrix until adsorption equilibrium is reached (126). Typically, the SPME headspace extracts of SCFAs in faecal samples followed by GC-MS analysis were performed on polyethylene glycol capillary columns (127). Novel studies deal with headspace SPME method performed with the use of divinylbenzene/Carboxen/polydimethylsiloxane (DVB/CAR/PDMS) fiber (126), or various commercially available fiber coatings, such as polydimethylsiloxane (PDMS), Carboxen/polydimethylsiloxane (CAR/PDMS), polyacrylate (PA), or polyethylene glycol (PEG) (128).

#### 4.1.4 Derivatization

Derivatization of samples is performed to improve the capability of compound identification and quantification. This procedure is typically done to change the analyte properties which leads to better separation and detection. Typically, the derivatization procedure can be combined with other extraction and concentration sample pretreatment techniques. The initial biological matrix is not always suitable for derivatization, and in some cases, pretreatment (a simple process of drying or a complicated process with more chemical reactions) is necessary to change such matrix (129). Further, it is demanded that the addition of a derivatization reagent will give a chemical reaction only with investigated analytes without affecting the matrix. Preferred chemical reactions for derivatization are i.) alkylation, ii.) silylation, iii.) acylation, iv.) addition to carbon-hetero multiple bonds, or v.) formation of cyclic compounds (130). The use of a suitable derivatization procedure depends on the physics-chemical properties of the analysed substances. In the case of acidic compounds, alkylation and silylation are the preferred approaches (130).

Silylation is the chemical reaction of replacing a reactive hydrogen atom in groups such as: -OH, -COOH, -SH, -NH, -CONH, -POH, and -SOH with the most frequently preferred reagent trimethylsilyl (TMS). The main purpose of this reaction is to change polarity, increase stability and improve separation behaviour. Alkylation is the formation of alkyl derivatives by the transfer of an alkyl group from one compound to another. Common alkylation of an acidic compound is performed by N, N-dimethylformamide dialkyl acetals. The formation of alkyl or aryl derivatives of acids results in esterification. This reaction is typically catalysed by strong acids and efficiency could be improved by removing water from the reaction (130). Various approaches are used to derivative the SCFAs and their selection usually depends on the separation technique used. GC and LC approaches are the preferred ones. A comprehensive overview of the common derivatization agents used in GC-MS and LC-MS approaches for SCFAs analysis in various biological samples is summarized in **Table 1**.

**Table 1:** Overview of the preferred derivatization reagents used in SCFAs analysis.

Matrix	Derivatization agent	Molecule	Method	Reference
Feces	DHPP	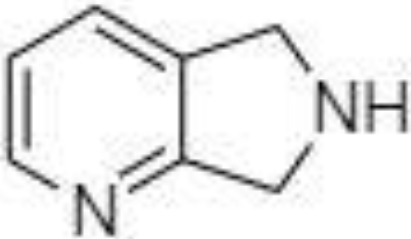	LC-MS/MS	(131)
	AMQ	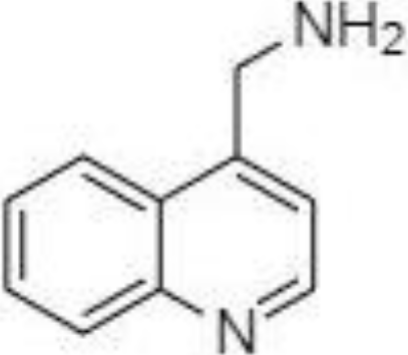	LC-MS/MS	(126)
	AABD-SH	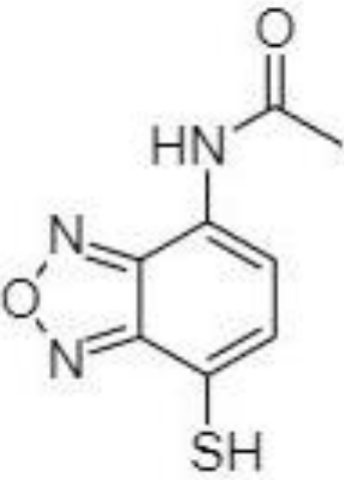	LC-MS/MS	(132)
	BP	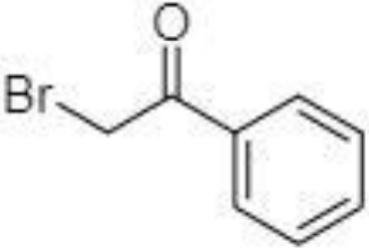	LC-MS/MS	(133)
	3-NPH	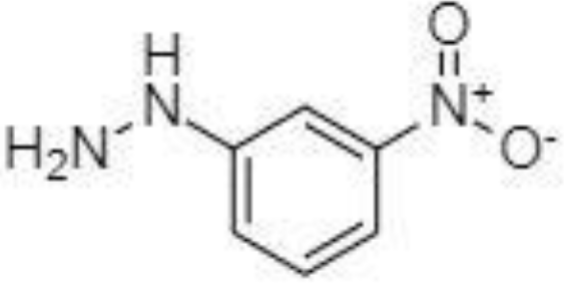	LC-MS/MS	(134)
	2-PA	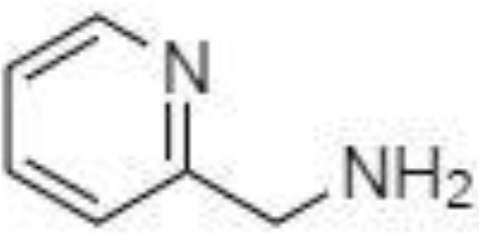	LC-MS/MS	(135)
	GT	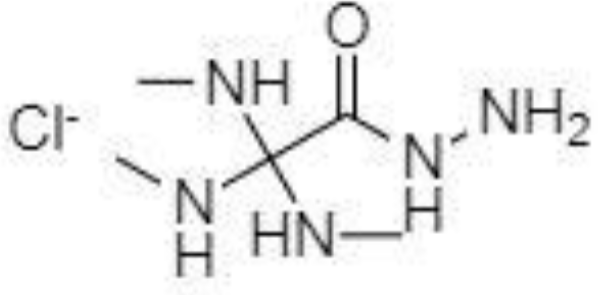	LC-MS/MS	(132)
	PCF	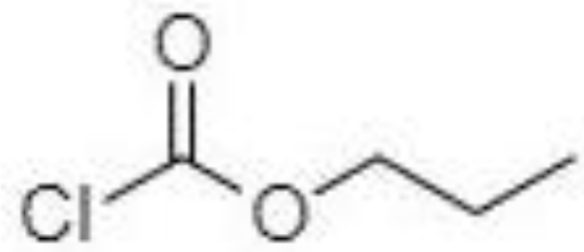	GC-MS	(136)
	BSTFA	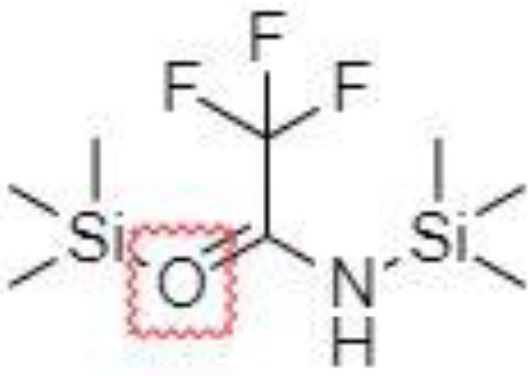	GC-MS	(47, 137)
	i-Bu	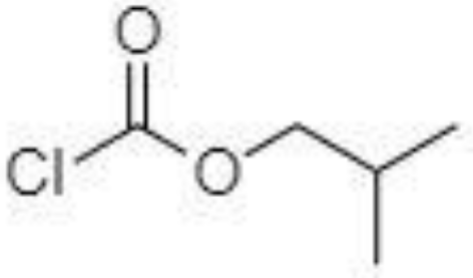	GC-MS	(138)
Serum	DHPP	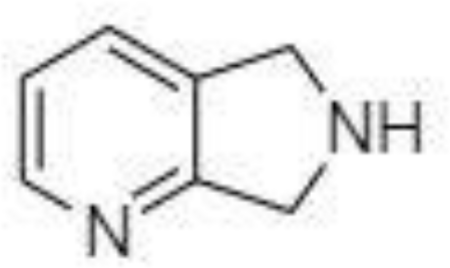	LC-MS/MS	(131)
	2-NPH	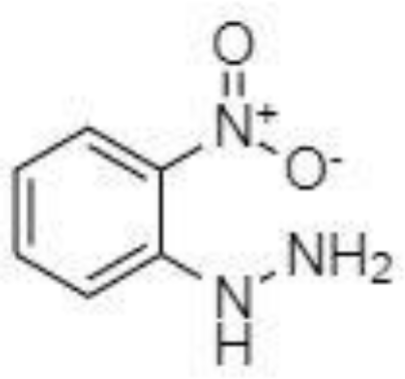	LC-MS/MS	(139)
	DIAAA	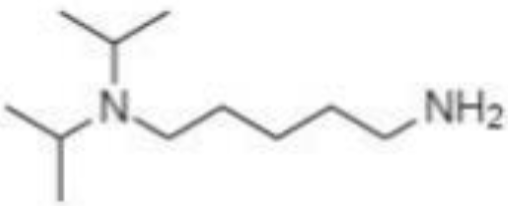	LC-MS/MS	(140)
Plasma	AABD-SH	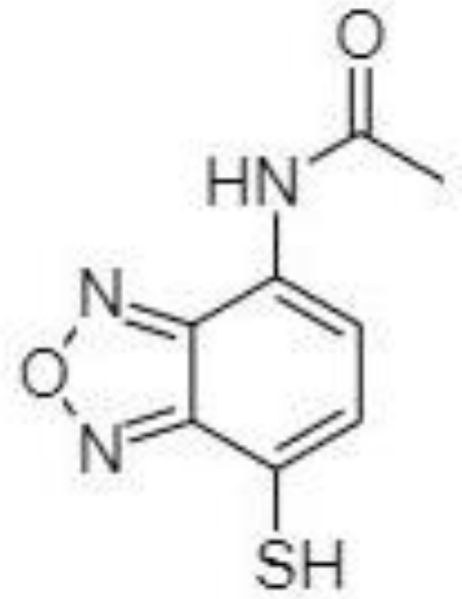	LC-MS/MS	(132)
	O-BHA	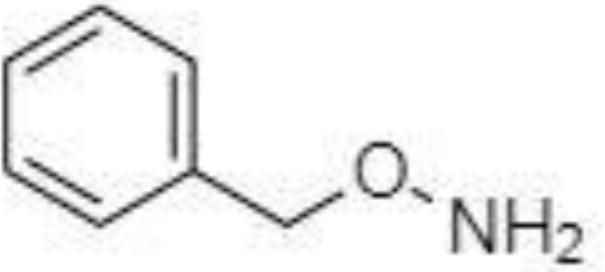	LC-MS/MS	(132, 141)
	BP	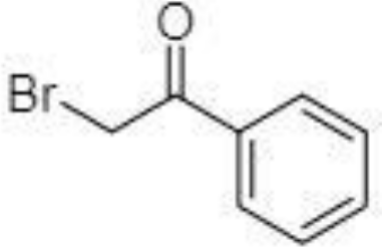	LC-MS/MS	(133)
	BSTFA	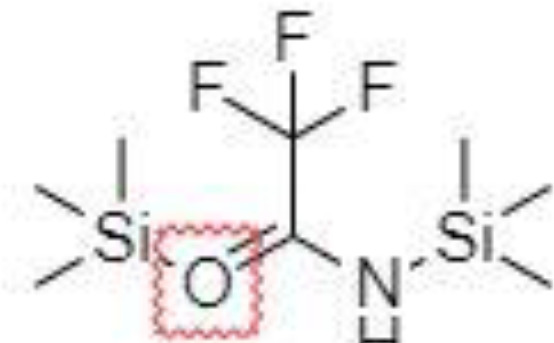	GC-MS	(47, 137)
Urine	O-BHA	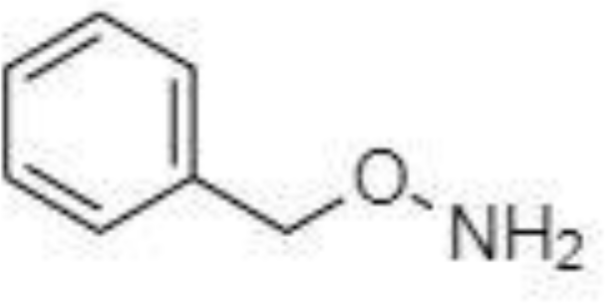	LC-MS/MS	(141, 142)
	BSTFA	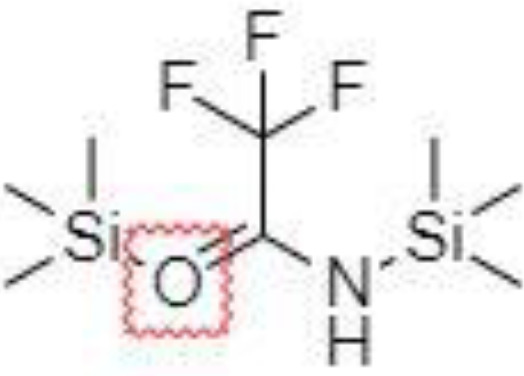	GC-MS	(47, 137)

2-NPH, 2-nitrophenylhydrazine; 3-NPH, 3-nitrophenylhydrazine; 2-PA, 2-picolylamine; AABD-SH, 4-acetamido-7-mercapto-2,1,3-benzoxadiazole; AMQ, 4-aminomethylquinoline; BP, 2-bromoacetophenone; BSTFA, N,O-bis(trimethylsilyl)trifluoroacetamide; DHPP, N,N-dimethyl-6,7-dihydro-5H-pyrrolo [3,4-d] pyrimidine-2-amine; DIAAA, 5-(diisopropylamino)amylamine; GT, Girard´s reagent T (trimethylaminoacetohydrazide chloride); i-Bu, isobutyl chloroformate; O-BHA, O-benzylhydroxylamine; PCF, propyl-chloroformate.

*Derivatization in GC*

Silylation derivatization procedure, and methyl-, ethyl-, propyl-, and isobutyl-acylation reagents are commonly used in GC-MS analysis of SCFAs (47, 126, 138, 143). Zheng et al. successfully applied propyl-chloroformate (PCF) derivatization which enabled the detection of nine SCFAs in various biological matrices – faeces, urine, and plasma. Such sample pretreatment procedure led to good sensitivity (limit of detection – LOD, levels under 100 pg) and linearity (136). Another derivatization procedure was developed for the determination of formic acid. The main problem associated with the analysis of formic acid with the use of GC is its coelution with the solvent peaks or instability of some derivate which affects the possibility to quantify such analyte in biological samples negatively. Therefore, pentafluorobenzyl bromide (PFBBr) was used to solve this discrepancy (144, 145).

Zhang et al. (47) presented an approach where the silylation with N, O-bis(trimethyl-silyl)-trifluoroacetamide (BSTFA) was applied to facilitate the separation and detection properties of nine SCFAs in faecal and serum samples. Such derivatization agent is very beneficial because the resulting derivatives are characterized by higher thermostability, volatility, and lower polarity in comparison to other reagents, which results in a wider range of injection and column conditions properties (146). However, BSTFA is highly sensitive to moisture and therefore, the samples must be dried. This fact improves the demands on the sample pretreatment (time, chemicals) procedure. On the other hand, such an approach is characterized by excellent recoveries, high repeatability and led to the analysis of SCFAs at low concentration levels (47).

*Derivatization in LC*

Direct analysis of SCFAs by LC-MS approach is practically impossible due to their very poor ionization efficiency. The main goal of the derivatization procedure before the own analytical step is the improvement of chromatographic behaviour and MS detection properties of the analytes. Typically, 3-nitrophenylhydrazine (3-NPH) is the preferred reagent used (134, 147). Han et al. (147) demonstrated excellent stability of the final products obtained with the use of 3-NPH – none of the derivatives showed observable degradation products for 3 months when storing at -20°C. Moreover, low LOD values, under 1 fmol, were obtained, which favours this procedure for routine biological sample pretreatment for SCFAs analysis.

The main SCFAs from C2 to C6 can be also detected in human faeces when derivatized with 2-nitrophenylhydrazine (2-NPH), 3-nitrophenylhydrazine (3-NPH) or using benzene ring containing reagents such as o-benzylhydroxylamine (O-BHA), or 2-bromoacetophenone (2-BP) (139, 142). The achievement of accurate SCFAs quantitation is typically obtained by the procedure based on the formation of the stable isotope-labelled versions of the NPH derivatives used as internal standards (147). However, such an approach is able to offer precise and accurate results, and the final costs of the analysis are significantly increased. The chemical reaction with 2-BP is fast and represents one of the most sensitive approaches used in SCFAs analysis. This fact was demonstrated by a 200 – 2000-fold higher sensitivity of the developed LC-MS method compared to the convenient GC-MS method (133).

**Figure 5:**
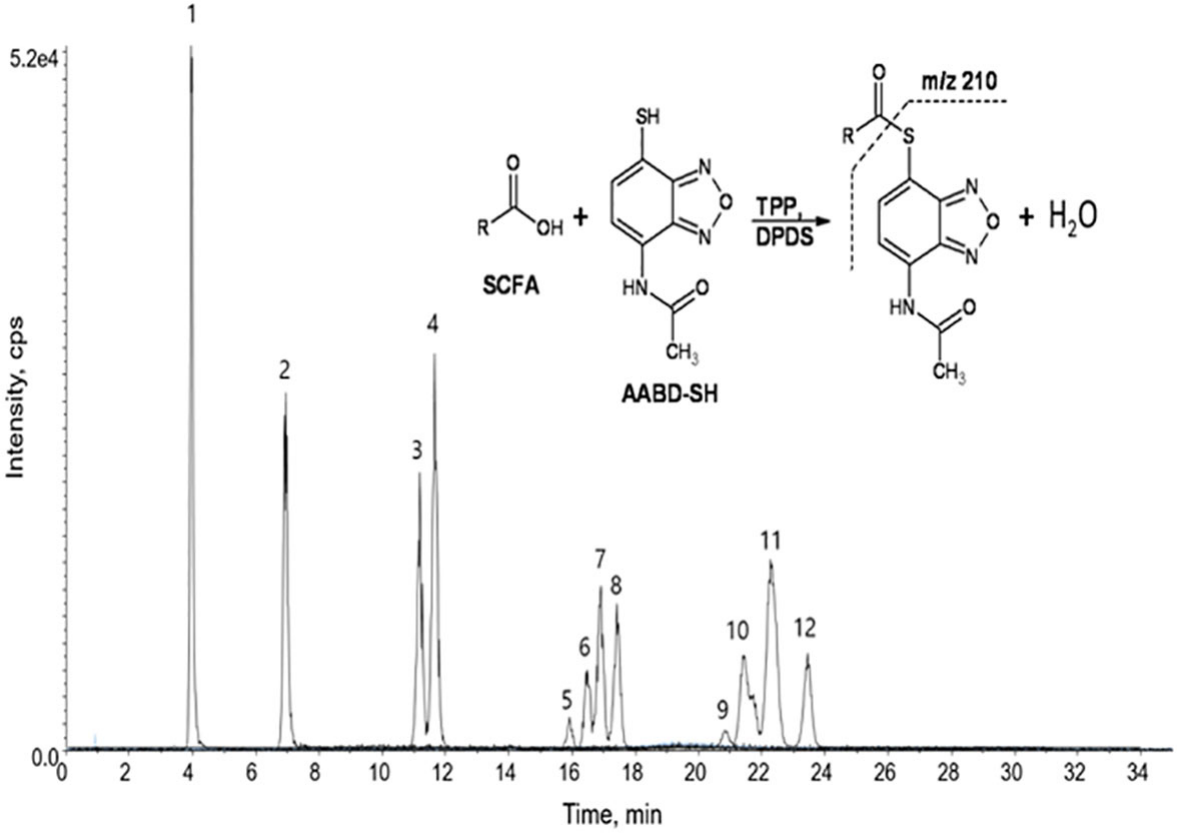
Extracted ion chromatogram of analysed SCFAs (1: acetic acid; 2: propionic acid; 3: isobutyric acid; 4: butyric acid; 5: 2,2-dimethylpropionic acid; 6: 2-methylbutyric acid; 7: isovaleric acid; 8: valeric acid; 9: 2,2-dimethylbutyric acid; 10: 2-ethylbutyric acid; 11: 2-methylvaleric acid; 12: caproic acid) and an illustrative scheme of derivatization procedure with the use of 4-acetamido-7-mercapto-2,1,3-benzoxadiazole (AABD-SH) and characteristic unique fragmentation pattern obtained during collision-induced dissociation (CID) in the MS/MS detection. Reprinted with permission from (132).

A very fast and simple derivatization procedure (5 min at room temperature) with the use of 4-acetamido-7-mercapto-2,1,3-benzoxadiazole (AABD-SH) prior to LC-MS/MS analysis was used by Song et al. (132). Characteristic fragmentation patterns and increased hydrophobicity after chemical derivatization enabled specific discrimination among investigated 12 SCFAs (see **Figure 5**) in various biological matrices – e.g., faeces, plasma, or human exhaled breath condensates.

Another derivatization possibility offers Girard’s reagent T (GT) - (carboxymethyl)trimethylammonium chloride hydrazide, which provides a permanent cationic charge of the molecules. Such an approach is beneficial because there is no need to add proton or sodium ion for ionization in ESI-MS (148). Excellent sensitivity improvement of the sample pretreatment procedure was described by Song et al. (149). The sensitivity of the GT derivatization was 1.2x10^8^ times higher than that of un-derivatized samples. Moreover, a 100-fold higher sensitivity was also observed when compared with the derivatization procedure based on the use of 3-NPH. According to these facts, the presented approach is more sensitive and does not demand the use of specific products such as isotopes which are in most cases very expensive.

#### 4.1.5 Comparison of the sample pretreatment procedures

Each sample pretreatment strategy used for the analysis of SCFAs in biological samples dispose with benefits and drawbacks. There is no universal sample preparation procedure which could be used. The selection of an appropriate approach is affected by the sample type and also by the final analytical technique used. The simplest procedures used in preparation of biological samples for SCFAs analysis include protein precipitation and LLE. There are no specific demands on instrumentation, the procedures are simple, relatively fast, economical and user friendly, and universal. As a limitation can be seen consumption of organic solvents which represent environmental burdens. Protein precipitation offers preparation of large amounts of samples and can be effectively adapted in an automatized manner. On the contrary, LLE is not well suited for the automatization, what can be very problematic especially from the clinical point of view because clinical environment demands high throughput procedures (process of large amounts of samples in short time).

Approaches based on SPE offer simple preconcentration and effective purification of the analytes. They are also characterized by high level of selectivity for targeted analyte or group of analytes what significantly improves accuracy and precision of the final analyses. Moreover, SPE can be performed also in micro format (µSPE) what significantly reduces demands on the biological sample and organic solvents necessary for elution of the analytes. The possibility to perform full automatized SPE procedure is also a great benefit.

Derivatization occupies a specific position in pretreatment of the samples for SCFAs analysis. It is an excellent tool to improve the separation or detection properties of the analytes. There is a large variety of chemical reagents which can be used to targeted modification of the investigated analytes. Moreover, typical derivatization reagents used for SCFAs structural modification are also very effective in derivatization of other non-volatile bacterial metabolites, such as lactate, which improves the amount of information obtainable from analysis of one sample.

### 4.2 Analytical approaches

The appropriate approach used for analysis of SCFAs typically depends on the purpose of the research and is conducted as a part of metabolomics. These molecules were investigated in various targeted and untargeted metabolomics strategies. Several analytical approaches have been developed for the accurate identification and quantification of SCFAs in biological samples – i.e., biological fluids (plasma, serum) and faeces. These approaches copy the preferred tools in metabolomics – i.e., nuclear magnetic resonance (NMR) and mass spectrometry (MS). NMR and MS strategies offer adequate analytical performance characteristics to achieve the demanded goal – identification of putative oncomarkers (150). Both crucial techniques offer their own advantages but have also specific disadvantages. NMR strategies are characterized by a lower sensitivity and selectivity in comparison to MS approaches. On the other hand, NMR requires minimal sample preparation and can be used as a non-invasive imaging of metabolites in cells and tissues (151–153). MS offers high sensitivity and selectivity, especially coupled with a separation technique (GC, LC, or CE). Such hyphenation in combination with an adequate sample preparation procedure is currently the most powerful analytical tool (150). It is also necessary to point out, that the content of SCFAs differs in accordance with the sample type analyzed. Their content in blood samples is much lower than that in faeces. Therefore, the analysis of blood samples is much more difficult and demands more sensitive approaches. Typically, the concentration of acetic acid in human plasma and serum samples ranges from 50 to 100 µM, propionic acid and butyric acid are present at a concentration range from 0.5 to 10 µM. The concentrations of other SCFAs are even lower (99).

#### 4.2.1 Gas chromatography

Gas chromatography (GC), coupled with various kinds of detectors (e.g., flame ionization detector – FID, MS), is the most used method of analysing organic acids in biological samples (154–157). GC-FID hyphenation represents a conventional approach used in SCFAs analysis. FID detection offers a wide spectrum of linearity and accuracy, inexpensive cost, and operation, as well as its ability to detect a wide range of concentrations of organic compounds (87). However, FID is a nonspecific detector that may not distinguish possible inferences when analyzing biological samples (158). Another drawback of this detection is the lack of additional information in the case of qualitative analysis. An effective tool to overcome the aforementioned drawbacks represents MS detection. The on-line GC-MS coupling results in enhanced sensitivity and selectivity, and the possibility to identify the known/unknown analyte unequivocally considering the presence of a large number of mass spectral libraries and databases. An overview of some most recent GC-MS methods used in the analysis of SCFAs in faeces and serum are summarized in **Table 2**.

**Table 2:** Overview of GC-MS methods used for analysis of SCFAs.

Matrix	Pretreatment	Stationary phase	Detection	LOD	LOQ	Reference
Feces/serum	Derivatization BSTFA	HB-5MS capillary column (30 m × 0.25 mm × 0.25 µm)	MS	0.064 – 0.067 μM	1.60 – 1.67 μM	(47)
Serum	Protein precipitation	Nukol (30 m × 0.25 mm× 0.25 µm)	MS	0,12 µM	–	(119)
Serum	LLE	DB-FFAP (Free Fatty Acid Phase) column (50 m × 0.32 mm, 0.5 μm)	MS	0.12 – 0.48 mg.L^-1^	0.40 – 1.61 mg.L^-1^	(100)
Faeces	LLE	Nukol (30 m × 0.25 mm× 0.25 µm)	MS	1.6 – 11.3 µg.mL^-1^	—	(159)
Plasma	Derivatization	DB-5MS capillary column (30 m × 0.25 mm × 0.25 μm)	MS	0.07 – 8.09 ng.mL^-1^	25 – 250 ng.mL^-1^	(101)
Faeces	Derivatization	HP-5 ms (5%-Phenyl-methylpolysiloxane) capillary column (30 m x 0.25 mm x 0.25 μm)	MS	0.002 – 0.01 µg.mL^-1^	0.02 – 0.09 µg.mL^-1^	(153)
	Acidification, extraction	Nukol (30 m × 0.25 mm× 0.25 µm)	MS	0.2 – 0.8 µg.mL^-1^	1 – 5 µg.mL^-1^	
Faeces/serum	LLE	Stabilwax^®^-DA (30 m × 0.25 mm × 0.25 μm)	MS	—	0.03 – 0.08 μM (serum) 4 – 50 μM (faecal water)	(160)
Faeces	Derivatization	DB-5ms DuraGuard (30 m × 0.25 mm × 0.25 μm)	MS	—	—	(161)
Serum	LLE	DB-FFAP column (30 m x 0.25 mm x 0.25 μm)	MS	0.1 – 0.6 µg.mL-1	—	(162)
Faeces	SPME	Supelcowax 10 capillary column (60 m x 0.32 mm)	MS	—	—	(84)
Plasma	LLE	DB-FFAP column (30 m x 0.25 mm x 0.25 µm)	MS	0.01 – 7.58 mg.L^-1^	0.03 – 22.75 mg.L^-1^	(163)
Faeces	Derivatization	1^st^ dimension: DB-225 ms (30 m × 0.25 mm × 0.25 μm) 2^nd^ dimension: DB-5 ms (30 m × 0.25 mm × 0.25 μm)	MS	—	—	(164)

—, not determined.

A rapid, convenient, and reliable method for SCFAs profiling in small amounts of mice faecal and serum samples by GC-MS approach using BSTFA in combination with sodium sulphate dehydration pretreatment was developed by Zhang et al. (47). The method was characterized by excellent linearity, accuracy, and sensitivity. The obtained LOD values were more favourable in comparison to some GC-MS approaches without derivatization procedure or GC-FID methods. The real application of the method confirmed increased levels of the major SCFAs (acetic acid, propionic acid, and butyric acid) associated with a high-fiber diet.

An effective GC-MS method without the need for a derivatization sample pretreatment procedure was developed for the analysis of SCFAs in the human serum of children with digestive disorders (100). The separation of 14 SCFAs was performed with the use of a high polar nitroterephthalic-acid-modified polyethylene glycol column (DB-FFAP) which is ideal for the analysis of volatile fatty acids without derivatization. The proposed method was comparable with other previously published GC approaches in terms of LOD and LOQ parameters. The application potential of this method was demonstrated by the measurement of samples from two different cohorts of children – subjects suffering from digestive diseases *vs.* healthy subjects. Higher serum SCFAs concentrations were observed in children with digestive disorders in comparison to healthy children. The developed approach by Wang et al. (100), used the same instrumental configuration as reported Zhang et al. (47). The difference is only in the separation column – Wang et al., used a longer column for better separations of 14 SCFAs while Zhang et al. performed the separation on a shorter column with thicker film for better sensitivity of 9 SCFAs. Both developed methods can be advantageously used for diagnostic purposes of the major SCFAs and their role in various diseases including cancer in the future.

In contrast, the method developed by Rahman et al. (119) separated 10 SCFAs in less than 12 min with simple protein precipitation before analysis. The used biological matrix in this method was serum and recovery levels were from 80 to 117% with superior sensitivity of 0.12 µM. The method was implemented in the analysis of samples obtained from obese and lean subjects. Significantly higher levels of acetic, propionic, isobutyric, butyric, isovaleric, and valeric acid were observed in obese subjects. Interestingly, a correlation has been found between SCFAs and waist circumferences which can indicate that levels of SCFA in serum are affected and may correlate with metabolic disorders.

Niccolai et al. (159) used a GC-MS approach to compare the faecal SCFAs profiles of celiac disease (CD), adenomatous polyposis (AP), and colorectal cancer (CRC) patients. The developed analytical approach was fast, and relatively simple, with appropriate accuracy and sensitivity. Significant changes were observed in the total SCFAs content of the CRC patients compared to healthy controls. The percentage composition of SCFAs was different in CRC and AP patients compared to healthy controls.

Human plasma samples from patients with chronic superficial gastritis (CSG), intestinal metaplasia (IM), and gastric cancer were also screened for SCFAs and tricarboxylic acid (TCA) cycle intermediates by a GC-MS method (101). A tetra-alkyl ammonium pairing procedure was used to prevent the loss of volatile SCFAs. Precision and accuracy results were within the ranges of 1.24–18.69% and 80.95–119.14%, and recoveries and matrix effects were within the ranges of 86.17–119.02% and 87.20–116.63%. Significantly decreased levels of propionic and butyric acid were investigated in cancer metabolic pathways. Moreover, some TCA cycle intermediates (such as cis-aconitate, α-ketoglutarate, and fumarate) were significantly decreased. Therefore, there is a potential that these molecules could be used to assess the progression of gastric cancer.

Cai et al. (153) compared two independent analytical platforms based on GC-MS and nuclear magnetic resonance (NMR) spectroscopy for SCFAs analysis. The whole procedure was realized with the use of mouse faecal samples, and parameters such as sensitivity, recovery, repeatability, or matrix effect were investigated and evaluated. Here, two GC-MS approaches were tested – the first one was accompanied by the sample preparation based on derivatization (propyl esterification), and the second one was represented by the analysis of underivatized samples. Superior sensitivity, recovery, and accuracy of SCFAs were exhibited by the GC-MS propyl esterification method. On the other hand, the NMR methods yielded better repeatability and minimal matrix effects compared to GC-MS methods. However, the NMR approaches suffer from low sensitivity. All methods generated good calibration curve linearity and comparable measurement of faecal SCFAs concentration.

Determination of free fatty acids (including SCFAs) as a tool for early detection of colorectal carcinoma (CRC) was performed by a GC-MS approach in plasma samples. An isotopic dilution GC-MS with a simple LLE before the determination of underivatized samples was characterized by appropriate accuracy and precision and sufficient LOD values (163). The method revealed significant changes of some SCFAs in colorectal carcinoma patients. Such panel of new plasma biomarkers would be useful for the identification of non-symptomatic subjects at risk to develop CRC and speed up the early CRC diagnosis, helping clinicians to correctly evaluate non-specific symptoms associated with various stages of the disease.

A comprehensive GC x GC-MS approach was used to analyse complex faecal metabolite (including SCFAs) changes in alcoholic liver disease (164). Such an approach utilizes two separation columns of differing phase selectivity and is characterized by improved sensitivity. Moreover, it allows the identification of a higher number of compounds. The analytical platform employed in this study successfully dissected the alterations of polar metabolites and SCFAs in faecal samples of the studied subjects.

Last but not least, GC-MS methods offer really sensitive analyses of SCFAs, with LOD values generally much lower than the natural levels of SCFAs in biological fluids and feces. Unequivocal identification and determination of the samples is supported by the large MS spectra libraries which are available for the GC-MS approach. Analysis that does not require derivatization is beneficial from a pretreatment perspective, for example, those conducted on stationary phases such as FFAP (free fatty acid phase). Moreover, the SPME and protein precipitation methods appear to be the most effective in reducing sample preparation time. It can be also stated that the GC-MS are universal and can be used in analysis of SCFAs in various types of samples and in investigation of various diseases.

#### 4.2.2 Liquid chromatography

Another method suitable for SCFAs analysis is liquid chromatography (LC) which is a very good alternative to GC methods. Typically, reverse phase (RP) LC approaches (with prior sample derivatization) are the preferred ones, and the LC-MS arrangement improves the sensitivity and selectivity of the determination. Moreover, the improvements in LC techniques (e.g., introduction of ultra-high-pressure liquid chromatography – UHPLC) led to significant shortening of the analysis time without compromising resolution. In general, the popularity of LC-MS analysis of body fluids is still increasing. It is due to the broad scale of advantages such as compatibility with the biological samples, high sensitivity, possibility to obtain a spectral information allowing analyte characterization (i.e., unequivocal confirmation of identity), ease of use, widespread availability in clinical laboratories and relatively modest cost per sample analysis (165, 166). The LC-MS approaches are more beneficial in comparison to GC-MS, because of minimization of the sample preparation procedure and broader application potential (167). A summary of some recent LC methods for SCFAs determination in various samples is presented in **Table 3**.

**Table 3:** Overview of LC methods used for analysis of SCFAs.

Matrix	Pretreatment	Stationary phase	Mobile phase	Detection	LOD	LOQ	Reference
Serum	Derivatization	RP mode, Ascentis® Express Phenyl-Hexyl column (50 mm × 2.1 mm, 2.7 μm)	A = 5 mM aqueous ammonium acetate B = isopropanol C = methanol Gradient elution Flow rate: 0.2 mL.min^-1^	MS/MS	—	5 pmol (FA 4:0) and 0.12 pmol (FA 6:0)	(102)
Feces	Liquid-liquid extraction	RP mode, Hypersil Gold aQ columns (150 mm x 4.6 mm, 3 and 5 µm)	A = 20 mM of NaH_2_PO_4_ (pH 2.2 adjusted with phosphoric acid) B = acetonitrile Gradient elution Flow rate: 1.25 mL.min^-1^	UV (210 nm)	0.13 – 0.23 mM	—	(168)
Feces	Derivatization	RP mode, Waters BEH C18 (100 mm x 2.1.mm, 1,7 μm)	A = water containing 0.01% formic acid B = acetonitrile containing 0.01% formic acid Gradient elution Flow rate: 0.35 mL.min^-1^	MS/MS	0.3 – 15 fmol	0.3 - 45 fmol	(147)
cell cultures of gut microbiota	Derivatization	HILIC mode, Zorbax HILIC plus column (100 mm × 4.6 mm, 3.5 μm)	A = water containing 20 mM ammonium acetate and 20 mM acetic acid B = acetonitrile Gradient elution Flow rate: 0.5 mL.min^-1^	MS/MS	0.001 – 30 fmol	0.01 – 30 fmol	(149)
Faeces, liver, kidney, brain, skeletal muscle, spleen, microbial fermentation media	Extraction	RP mode, Hypercarb – porous graphitic carbon, PGC (50 mm × 2.1 mm, 3 μm)	A = water containing 0.1% formic acid B = acetonitrile Gradient elution Flow rate: 0.15 mL.min^-1^	MS/MS	0.001 – 0.003 mM	0.003 – 0.009 mM	(48)
Plasma, urine	Derivatization, LLE	RP mode, Kinetex Evo C18 column (50 mm × 2.1mm, 1.7 μm)	A = water containing 0.1% formic acid B = methanol Gradient elution Flow rate: 0.6 mL.min^-1^	MS/MS	—	25.2 ng.mL^-1^ (LLOQ)	(141)
Serum	Derivatization	RP mode, Restek Raptor C18 (100 mm x 2.1 mm, 2.7 μm)	A = water containing 0.1% formic acid B = acetonitrile Gradient elution Flow rate: 0.4 mL.min^-1^	MS/MS	3 ng.mL^-1^	10 ng.mL^-1^	(120)
Serum	Derivatization, extraction	RP mode, BEH C18 (100 mm × 2.1 mm)	A = water B = acetonitrile Gradient elution Flow rate: 0.3 mL.min^-1^	MS/MS	—	0.00025 – 2.5 µM	(169)
Plasma, exhaled breath condensate	Derivatization	RP mode, Pursuit 5 C18 (150 mm × 2.0 mm)	A = water containing 0.1% formic acid B = acetonitrile containing 0.1% formic acid Gradient elution Flow rate: 0.5 mL.min^-1^	MS/MS	—	0.158 – 1.03 µM	(132)
Serum	Derivatization, extraction	RP mode, ACE C18-PFP (100 mm x 2.1 mm, 1.7 μm)	A = water B = acetonitrile Gradient elution Flow rate: 0.3 mL.min^-1^	MS/MS	1 – 7 ng.mL^-1^	3 – 19 ng.mL^-1^	(170)
Faeces	Derivatization	RP mode, Kinetex XB-C18 (50 mm × 2.1 mm, 2.6 µm)	A = water containing 0.1% formic acid B = acetonitrile containing 0.1% formic acid Gradient elution Flow rate: 0.25 mL.min^-1^	MS/MS	0.03 – 1.9 µmol.g^-1^	—	(134)
Serum, colon content	Derivatization	RP mode, Waters Acquity BEH C18 c (50 mm × 2.1 mm, 1.7 µm)	A = water containing 0.01% formic acid B = acetonitrile containing 0.01% formic acid Gradient elution Flow rate: 0.3 mL.min^-1^	MS/MS	—	—	(171)
Bovine milk and serum	Derivatization	RP mode, Atlantis PREMIER BEH C18 AX (150 × 2.1 mm, 1.7 µm)	A = water containing 0.1% formic acid B = acetonitrile containing 0.1% formic acid Gradient elution Flow rate: 0.2 mL.min^-1^	MS/MS	0.0323 – 0.17 µM	0.1 – 0.55 µM	(172)
Faeces, serum, urine, duodenal content	Derivatization	RP mode, CSH C18 (100 mm × 2.1 mm, 1.7 μm)	A = water containing 0.1% formic acid B = acetonitrile containing 0.1% formic acid Gradient elution Flow rate: 0.4 mL.min^-1^	MS/MS	25 nM	50 nM (LLOQ)	(173)

—, not determined.

Chen et al. (102) developed an LC-MS/MS method for SCFAs which was applied to the serum samples obtained from Japanese volunteers. The optimized LC-MS/MS method for NPH derivatives of the analyzed fatty acids (FA) was relatively fast (total time of analysis was less than 10 min) and showed favorable validation parameters. The coefficient of variation of reproducibility and recovery for FA 4:0, was 3.1% and 5.3%, respectively, and for FA 6:0 the value of 6.1% and 3.0% was obtained. The average recoveries were 94.7 ± 5.0% (FA 4:0) and 96.1 ± 2.9% (FA 6:0). It was demonstrated that the developed method is accurate for the analysis of free and also esterified SCFAs in human serum and may be used for their monitoring in large varieties of clinical samples.

The choice of appropriate LC methods for SCFAs analysis of fecal samples depends on the purpose of the analysis and on the expected concentration of the analytes present in the sample. Therefore, less sensitive LC approaches can be also used for the expected purpose. For example, De Baere et al. (168) presented an LC method in combination with simple UV detection which was used for the determination of four SCFAs (formic, acetic, propionic, and butyric acid) in supernatants of fecal bacterial cultures. The simple sample preparation (LLE) and no derivatization step make the method cost-effective and simple. Moreover, the total time of analysis was 15 min. Although the method offers significant benefits, its application on real samples of body fluids or faeces is disputable. The main application limiting step of such methods is their selectivity and sensitivity. As a good example can be used the paper published by Han et al. (147) which deals with the LC-MS/MS approach. Both methods – i.e., LC-UV and LC-MS/MS provided the separation on a reversed-phase column, but the sensitivity of the LC-MS/MS approach was much higher. The LC-MS/MS method by Han et al. was equipped with UHPLC separation system which enabled baseline resolution of all 10 analyzed SCFAs including isomers C_4_, C_5_, and C_6_. The use of ^13^C-labeled IS was beneficial due to the elimination of matrix effects on the quantitation measurements of the SCFAs in human fecal samples (147).

A completely different approach was introduced by Song et al. (149) who used HILIC stationary phase and GT derivatization procedure. The developed approach was used to quantitate SCFAs from a small volume of total extracellular metabolites produced by *Eubacterium rectale*. GT derivatization of SCFAs developed in the study showed excellent linearity, and also better sensitivity than approaches without derivatization or with chemical derivatization. Such a method has a good assumption to be used as a rapid and high-throughput screening platform for SCFAs in various types of samples.

**Figure 6:**
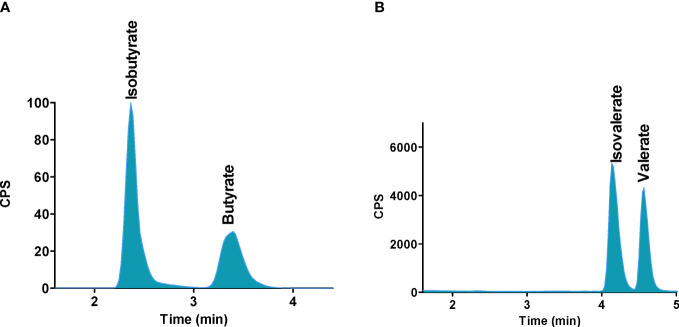
Separation of SCFAs isomers and stereoisomers with the use of PGC column. **(A)** Isobutyrate and butyrate, **(B)** Isovalerate and valerate. Reprinted with permission from (48).

A LC-MS/MS technique for the analysis of SCFAs in tissues and biological fluids without derivatization using isotope labelled internal standards was recently developed (48). Typically, the LC-MS approach, similar to GC-MS, requires laborious multi-step sample pretreatment which includes sample filtration and derivatization. Here, the presented LC-MS method was characterized by fast and simple sample preparation and short LC runtime (10 min). The use of Hypercarb PGC separation column which is stable at all pH ranges (0–14), high temperatures, and aggressive mobile phases was beneficial in the case of isomers separation (isobutyrate and butyrate, isovalerate and valerate). An illustrative record of separated SCFAs isomers is presented in **Figure 6**. Isomeric SCFAs could not be separated using a normal silica base C18 column without derivatization. Baseline chromatographic separation with an appropriate resolution of these compounds is necessary for their accurate quantification because the isomers have the same molecular and fragmentation ions (butyrate and isobutyrate) or the same parent and fragmentation ions (isovalerate and valerate). After a successful validation procedure, the method was applied for the determination of SCFAs in a large variety of biological samples – i.e., mouse and human faecal and mouse liver, kidney, brain, skeletal muscle, spleen samples, and microbial fermentation media. The authors stated, that the only one limitation of the method is its lower recovery in the case of some substances when analyzing the brain, liver, and plasma samples. This fact may be caused by a matrix effect associated with such types of samples.

Jaochico et al. (141) introduced a LC-MS method with derivatization and following LLE for absolute quantitation of nine SCFAs using stable isotope-labeled internal standards. Optimised derivatization, sample preparation, and C18 reversed-phase LC–MS/MS conditions produced a relatively clean sample with baseline chromatographic resolution. However, the originally developed method was not suitable for the quantification of acetic acid in the samples. Modification in the extraction protocol (the use of dichloromethane instead of ethyl acetate) led to the successful quantification of this compound.

An interesting study was performed by Dei Cas et al. (120) who compared their newly developed LC-MS method for SCFAs analysis with a convenient GC-MS approach. It was demonstrated that the GC-MS approach was accompanied with very tedious and long-timing sample preparation. Moreover, a relatively large amount (500 µL) of the biological sample (serum) was required for the analysis. The approach of LC-MS was driven by a shorter time of analysis and sample preparation. Due to the high cost of labeled analogues of SCFAs accompanied by an inherent difficulty in their chemical synthesis, different affordable options were evaluated. Finally, 2-isobutoxyacetic acid was used as a suitable IS for quantification purposes. The method was applied to investigate the physiological ranges of SCFAs and medium-chain fatty acids in serum obtained from a heterogeneous healthy population (from 18 to 85 years old). The following concentration ranges were found: 935.6 ± 246.5 (butyric), 698.8 ± 204.7 (isobutyric), 62.9 ± 15.3 (valeric), 1155.0 ± 490.4 (isovaleric) and 468.7 ± 377.5 (hexanoic) ng.mL^-1^ respectively (mean ± SD).

An effective solution in identification and quantification of large variety of metabolites (including SCFAs) with the use of the LC-MS approach is represented by the Orbitrap based MS step. As a very good examples, the recently published papers by Gardana et al. (174) or Chen et al. (175) can be mentioned. The implementation of such high resolution MS detection offers the possibility to analyze the demanded molecules according to their exact mass what minimizes the possible mistakes during their quantification in complex biological samples – e.g. false positive results, overlapping and co-eluting peaks. The main disadvantage of such approach is the price of the instrument and demands on the highly skilled personal.

In general, LC-MS approaches represent very fast, robust and sensitive analytical tool for the analysis of SCFAs in biological matrices. As mentioned previously, the advanced UHPLC instrumentation contributed to the significant shortening of the total running time without the loss of the resolution and enabled high sample throughput.

#### 4.2.3 Capillary electrophoresis

Capillary electrophoresis (CE) is a powerful and versatile technique that can be used to separate a wide range of analytes – inorganic ions, small molecules, peptides, and also large proteins. CE represents an effective alternative and/or complement approach to the preferred HPLC and GC techniques. However, CE is characterized by a large variety of benefits such as low sample and organic solvent consumption, work in aqueous environments resulting in eco-friendly aspects of analysis, high separation efficiency, and relatively short time of analysis, it suffers from poor concentration limits (176). Therefore, various preconcentration strategies realised directly in the separation capillary, and coupling with more sensitive and selective detection techniques (especially MS) are implemented into the routine CE analyses. Significant improvements in CE-MS coupling led to the fact that CE is an established tool used in metabolomics or proteomics (177–180). It is often applied in the metabolic profiling of various body fluids. However, there are only few papers dealing with the analysis of SCFAs by CE, and according to our best knowledge only one experimental work was realised with the use of CE-MS. A comprehensive overview of the most recent CE methods for SCFAs is presented in **Table 4**.

**Table 4:** Overview of CE methods used for analysis of SCFAs.

Matrix	Pretreatment	Separation capillary	BGE	Detection	LOD	LOQ	Ref.
Feces	Aqueous extraction	Silica capillary coated with polyacrylamide, 37 cm total length, 50 μm ID	234 mM phosphate buffer (pH 6.10) with 12% (v/v) methanol	UV (200 nm)	–	16.1 -49.6 µM	(181)
Faeces (mice)	Acidification, aqueous extraction	Fused silica capillary, 55 cm effective length, 60 cm total length, 75 um ID	160 mM tris(hydroxymethyl)aminomethane + 10 mM benzoic acid (pH 8.5)	Indirect UV (228 nm)	0.03 – 0.19 mM	0.09 – 0.43 mM	(182)
Faeces (human, canine)	Aqueous extraction	Fused-silica capillary, 71.5 cm effective length, 80 cm total length, 75 um ID	10 mM β-alanine + 10 mM benzoic acid (pH 5.7) + 4 mM α-CD + 0.005% polybrene	Indirect UV (350 nm)	—	8 – 10 μM	(183)
Faeces (rat)	Aqueous extraction	Fused-silica capillary, 56 cm effective length, 64.5 cm total length, 75 um ID	5 mM α-CD, 8 mM 5’-AMP, 7.5% (v/v) MeOH, 100 mM boric acid (pH 6.5)	Indirect UV (320 nm)	—	—	(184)
Feces	Liquid-liquid extraction, Lyophilization	Fused-silica capillary, 49 cm effective length, 58 cm total length, 50 um ID	0.5 mM tetradecyl trimethyl ammonium bromide (TTAB) + 10 mM 1,3(6,7)-naphthalenetrisulfonate (NTS) + 5.0 mM 1,5-naphthalenedisulfonic acid (NDS) + 5.0 mM sodium chromate + 10 mM of diethylamine (DEA) (pH 11.5)	Indirect UV (280 nm)	1.2 – 3.6 μM	4 – 12 μM	(185)

The older experimental works dealing with the CE analysis of some SCFAs published before the year 2000 were typically performed under normal polarity separation condition with indirect UV or fluorescence detection in matrices such as serum (186) or saliva (187). CE-MS approach was used for analysis of propionic acid in urine samples (188).

Garcia et al. introduced a CE-UV method for analysis of short-chain organic acids (including four SCFAs) based on separation in a polyacrylamide coated capillary whereas the separation medium was composed of 234 mM phosphoric acid (adjusted to pH 6.1 with NaOH) and 12% methanol. The developed method was compared with an orthogonal ion chromatography (IC) approach. The analysis of four SCFAs was approximately two-times faster in comparison to the IC method. CE-UV offered the limit of quantification (LOQ) values from 16.1 to 49.6 µM for investigated four SCFAs. Moreover, the method was characterized as fast and simple, sample pretreatment demanded only aqueous extraction with further direct injection into the CE analyser. Finally, the validated method was applied to analyse 136 faecal samples of Mediterranean elderly people (181).

A rapid and low-cost CE method with indirect UV detection for the determination of three SCFAs in mice faeces was introduced by Marques et al. (182). The method was applied to identify SCFAs concentration changes in mice with dextran sulfate sodium-induced colitis undergoing the treatment with *Plinia caulifora* aqueous extract.

A CE method with indirect UV detection was also introduced by Hodek and Krizek (183). The authors used a background electrolyte (BGE) composed of a mixture of β-alanine and benzoic acid with two additives – i.e., polybrene (necessary for reversal of the electroosmotic flow – EOF), and α-cyclodextrin, α-CD, (necessary for separation of butyric and isobutyric acid. The addition of polybrene, a positively charged surfactant, into the BGE resulted in a shorter analysis time. Pentanesulfonic acid was used as an internal standard. The developed method was used to quantify selected SCFAs in human and canine faeces samples with the following concentration levels: i) human samples: acetic acid (276.5 ± 3.7 μg.g^-1^), propionic acid (104.3 ± 3.7 μg.g^-1^), isobutyric acid (10.8 ± 3.5 μg.g^-1^), butyric acid (107.7 ± 11.3 μg.g^-1^); ii) canine samples: acetic acid (777.7 ± 71.5 μg.g^-1^), propionic acid (300.9 ± 27.4 μg.g^-1^), isobutyric acid (34.9 ± 9.8 μg.g^-1^), butyric acid (204.1 ± 19.0 μg.g^-1^).

A similar strategy based on CE with indirect UV detection and the use of α-CD as a constituent of the BGE was introduced by Pham et al. (184) for the determination of SCFAs and medium-chain fatty acids in rat faecal samples. Here, 5’-adenosine mono-phosphate (5’-AMP) was used as an indirect photometric detection reagent. Various separation conditions were investigated to obtain appropriate resolution, efficiency, and signal-to-noise ratio levels. Finally, the method was applied to determine the concentrations of selected fatty acids in rat faeces.

CE with indirect UV detection was also used for the determination of SCFAs and some inorganic ions in faecal samples of paediatric inflammatory bowel disease patients. The sample handling was based on a lyophilization procedure which facilitates the whole extraction process. The developed method was applied to a cohort of children with Crohn’s disease and ulcerative colitis treated with exclusive enteral nutrition or corticosteroid therapy.

An innovative approach based on paired ion electrospray ionization MS (PIESI) coupled with CE was presented in 2017 (189). PIESI is a common procedure suitable for the analysis of anionic compounds in positive MS mode. Anionic molecules (SCFAs) are able to form anion/ion-pairing reagent complexes through the process based on the addition of multiple charged reagents by continuous infusion after separation. Then, the positively charged complexes are detected using positive ESI mode with high sensitivity and stability (190). Such a technique was applied to determine saturated fatty acids, including SCFAs, by using dicationic ion-pairing reagents (here N,N’-dibutyl 1,1’-pentylenedipyrrolidium) forming singly charged complexes with anionic analytes. However, the authors declared good linearity and LOD values ranging from 0.37 to 2.88 µg.mL^-1^, the method was applied only for the analysis of food (cheese and coffee) samples. It is expected that the method after some modifications could be also implemented into the analysis of biological fluids and tissues (189).

#### 4.2.4 Comparison of the analytical methods

According to the overview of analytical methods used for determination of SCFAs summarized in **Tables 2** – **4**, it can be stated that GC-MS and LC-MS approaches are the dominant ones in that field. In case of GC-MS methods it was possible to separate up to 14 various SCFAs. The summarized approaches also declare that in some cases the developed methodology was implemented not only in the analysis of SCFAs but also into the simultaneous determination of other metabolites – e.g. methodology used by Kim et al. (101). So, such approaches can be effectively used in screening of larger variety of endogenous metabolites. The GC-MS methods were able to offer LOD values at ng.mL^-1^ concentration levels what reflects very good sensitivity of such experimental approach. Very similar performance parameters (e.g. time of analysis, LODs) and separation possibilities (number of analysed SCFAs and other metabolites) were also obtained in case of LC-MS methodologies. However, LC-MS approaches were used in much broader application range – samples such as urine (141), cell cultures (149), and exhaled breath condensate (176) were analysed. Therefore, the LC-MS approach can be characterized by high level of versatility. Moreover, LC offers possibilities for simple on-line sample preparation or development of methodologies based on so called “hearth-cut” approach. Such methodologies can be effectively used in targeted analysis of highly specific analytes present in the sample at very low concentration and/or selection of narrow range of analytes which separation is relatively problematic.

As can be seen, CE still play the role of an alternative or comparative method to the dominant chromatographic methods. The CE approach was applied only on the faeces samples and the obtained LOD values were worse as in the case of LC or GC. Moreover, the CE analyses focused only on small number of analysed SCFAs. We expect, that in a near future the on-line combination of CE with MS will be used to improve the potential of electromigration methods in SCFAs analysis. However such hyphenation could bring similar LOD values as in case of chromatographic methods, we do not expect that the CE-MS combination will replace the chromatography approaches.

## 5 Conclusions

The gut microbiota plays a crucial role in many physiological and also pathological processes in the human body. Its dysbiosis can alter metabolism and immune responses which results in the development of various diseases, including cancer. SCFAs are metabolites produced by microorganisms in our digestive tracts. These molecules have demonstrated a certain connection with cancer diseases, and it is suggested that they can serve as putative biomarkers or therapeutic modalities. Various oncological diseases may be reliably characterized by SCFAs what can bring a revolution to clinical pharmacology or oncology. Since cancer biomarkers have become more diverse, SCFAs can serve not only as biomarkers of disease but also estimate the effectiveness of cancer therapy. Recently, the identification and/or determination of metabolic biomarkers are typically carried out by advanced analytical approaches based on gas chromatography (GC), liquid chromatography (LC), or capillary electrophoresis (CE) and their connection with MS detection. Such sophisticated approaches are highly demanded especially in the clinical monitoring of patient samples, due to their high degree of selectivity, accuracy, and sensitivity. It is suggested that the aforementioned analytical tools will play a crucial role in the establishment of reliable cancer biomarkers (including individuals or a panel of SCFAs) in the near future. Moreover, we expect that novel MS strategies based e.g. on MALDI imaging will be implemented into the clinical environment, and will play a significant role in a detailed understanding of various diseases (not only cancer) and led to their early diagnosis, overcoming, and treatment.

## Author contributions

All authors were involved in the conception of the manuscript, the drafting, and/or critical reviewing of the manuscript, and have approved the final version for publication.
